# Pregnancy-Specific Glycoproteins Bind Integrin αIIbβ3 and Inhibit the Platelet—Fibrinogen Interaction

**DOI:** 10.1371/journal.pone.0057491

**Published:** 2013-02-28

**Authors:** Daniel K. Shanley, Patrick A. Kiely, Kalyan Golla, Seamus Allen, Kenneth Martin, Ronan T. O’Riordan, Melanie Ball, John D. Aplin, Bernhard B. Singer, Noel Caplice, Niamh Moran, Tom Moore

**Affiliations:** 1 Department of Biochemistry, University College Cork, Cork, Ireland; 2 Centre for Research in Vascular Biology, University College Cork, Cork, Ireland; 3 Department of Life Sciences, and Materials and Surface Science Institute, University of Limerick, Limerick, Ireland; 4 Molecular & Cellular Therapeutics, Royal College of Surgeons in Ireland, Dublin, Ireland; 5 Maternal and Fetal Health, Manchester Academic Health Sciences Centre, University of Manchester, Manchester, United Kingdom; 6 Institute of Anatomy, University Hospital Essen, Essen, Germany; Otto-von-Guericke University Magdeburg, Germany

## Abstract

Pregnancy-specific glycoproteins (PSGs) are immunoglobulin superfamily members encoded by multigene families in rodents and primates. In human pregnancy, PSGs are secreted by the syncytiotrophoblast, a fetal tissue, and reach a concentration of up to 400 ug/ml in the maternal bloodstream at term. Human and mouse PSGs induce release of anti-inflammatory cytokines such as IL-10 and TGFβ1 from monocytes, macrophages, and other cell types, suggesting an immunoregulatory function. RGD tri-peptide motifs in the majority of human PSGs suggest that they may function like snake venom disintegrins, which bind integrins and inhibit interactions with ligands. We noted that human PSG1 has a KGD, rather than an RGD motif. The presence of a KGD in barbourin, a platelet integrin αIIbβ3 antagonist found in snake venom, suggested that PSG1 may be a selective αIIbβ3 ligand. Here we show that human PSG1 binds αIIbβ3 and inhibits the platelet – fibrinogen interaction. Unexpectedly, however, the KGD is not critical as multiple PSG1 domains independently bind and inhibit αIIbβ3 function. Human PSG9 and mouse Psg23 are also inhibitory suggesting conservation of this function across primate and rodent *PSG* families. Our results suggest that in species with haemochorial placentation, in which maternal blood is in direct contact with fetal trophoblast, the high expression level of PSGs reflects a requirement to antagonise abundant (3 mg/ml) fibrinogen in the maternal circulation, which may be necessary to prevent platelet aggregation and thrombosis in the prothrombotic maternal environment of pregnancy.

## Introduction

Pregnancy-specific glycoproteins (PSGs) are expressed throughout human pregnancy and are the most abundant fetal proteins secreted by the placental syncytiotrophoblast into the maternal bloodstream in mid to late pregnancy (∼200 - 400 µg/ml) [1]–[3]. PSGs are produced by ten *PSG* genes (*PSG1– PSG9, PSG11*) and belong to the carcinoembryonic antigen (CEA) family, part of the immunoglobulin (Ig) superfamily [4]. Human PSG proteins consist of four immunoglobulin (Ig)-like domains – an amino-terminal Ig variable-like (N) domain and three Ig constant-like (C) domains and, like the closely related CEACAMs, are heavily N-glycosylated, particularly on the amino-terminal N-domain [5]. The mouse has seventeen *Psg* genes (*Psg16*– *Psg32*) that are expressed by the trophoblast giant cells and the spongiotrophoblast. The domain structure of mouse Psg proteins is more diverse than human PSGs but the majority have three Ig variable-like N domains (N1 - N3) and one carboxy-terminal Ig constant-like (C) domain. The lack of discernible orthologous relationships between primate and rodent *PSG*s suggests that these gene families evolved independently from a common ancestor in the different mammalian lineages, suggesting strong selection pressure driving rapid evolution [6]. However, notwithstanding the lack of orthology and the different domain contents of human and mouse PSGs, they share conserved functions, as outlined below. Moreover, the human PSG N-domains and the mouse N1-domains exhibit evidence of conserved or convergent evolution [6].

Studies of PSG function have largely focussed on their role in modulating the maternal immune system. PSGs isolated from the human placenta have an inhibitory effect on phytohaemagglutinin or allogeneically stimulated lymphocytes [7], [8]. Subsequently, it was shown that recombinant mouse and human PSGs induce production of anti-inflammatory cytokines such as interleukin-10 (IL-10) and transforming growth factor beta-1 (TGFβ1) by monocytic, macrophage and dendritic lineages in vitro and in vivo [9]–[14]. In the human, elevated PSG levels are associated with improved symptoms of rheumatoid arthritis during pregnancy [15]. These findings are consistent with PSGs contributing to modulation of maternal immune responses during pregnancy. More recently, PSGs were shown to be pro-angiogenic in *in vitro* assays, an activity mediated by interactions with cell surface glycosaminoglycans and the induction of TGFβ1 and vascular endothelial growth factor A (VEGF-A) [16]–[18]. PSGs are expressed from the preimplantation blastocyst stage of development [19] and therefore may have a role in promoting angiogenesis in the placental bed in the early pregnancy, or perhaps in vascular endothelial protection and repair in the maternal circulation in later pregnancy. Consistent with the proposed immunoregulatory and angiogenic functions of PSGs, deregulation of PSG expression has been reported in disorders of pregnancy associated with pro-inflammatory and anti-angiogenic phenotypes. For example, low levels of PSGs have been reported in maternal circulation of first and second trimester pregnancies complicated by intrauterine growth retardation and preeclampsia [20], [21].

Relatively little is known about the cellular receptors that underpin PSG functions. The tetraspanin CD9 is a receptor for mouse Psg17 and Psg19, but not mouse Psg23 or human PSG1, an interaction that requires N-glycosylation of the PSG protein N-terminal domain [13], [22]. Human PSG1 and mouse Psg17 and Psg23 bind cell surface proteoglycans such as syndecans and glypican-1; again, this interaction appears to be influenced by PSG protein N-glycosylation because binding activity is influenced by the cell type used to produce recombinant PSG proteins [23].

The presence of the tripeptide motif Arg-Gly-Asp (RGD) on an exposed loop of the N-domain of most human PSG proteins suggests that at least some of their functions may be mediated through integrin binding [6]. Snake venoms contain disintegrin proteins that bind integrins and disrupt cell - extracellular matrix interactions or blood clotting mechanisms and, analogously, we and others have hypothesised that PSGs may function as secreted integrin ligands that disrupt integrin function and thereby facilitate invasion of maternal tissues by fetal trophoblast, or disrupt other integrin mediated functions in maternal tissues [6], [24]. We noted that PSG1 has a KGD rather than an RGD motif and, based on the presence of a KGD motif in barbourin, a platelet integrin αIIbβ3 antagonist found in pygmy rattlesnake (*S. m. barbouri*) venom [25], we tested whether PSG1 may be a selective αIIbβ3 ligand. In this report we show that, like barbourin, human and mouse PSGs bind αIIbβ3 and inhibit the platelet – fibrinogen interaction.

## Results

We previously showed that a small number of mouse *Psg* genes produce the bulk of Psg mRNA and that *Psg22* alone accounts for more than 90% of Psg mRNA in the first half of pregnancy [26]. To determine whether human *PSG* genes exhibit similarly pronounced disparities in expression we used three sets of redundant primers and combined RT-PCR, with cloning and sequencing of PCR products, and examined relative expression of *PSG* genes in placentas obtained at first trimester and term. Similar to the mouse, we found that a small number of PSGs produced the bulk of PSG mRNA, with *PSG1* exhibiting high relative expression throughout gestation (Fig. S1 in File S1). These results were confirmed by analysing published RNA-Seq data from human term placentas [27], [28], in which PSG1 was the most abundant *PSG* gene transcript (data not shown).

**Figure 1:**
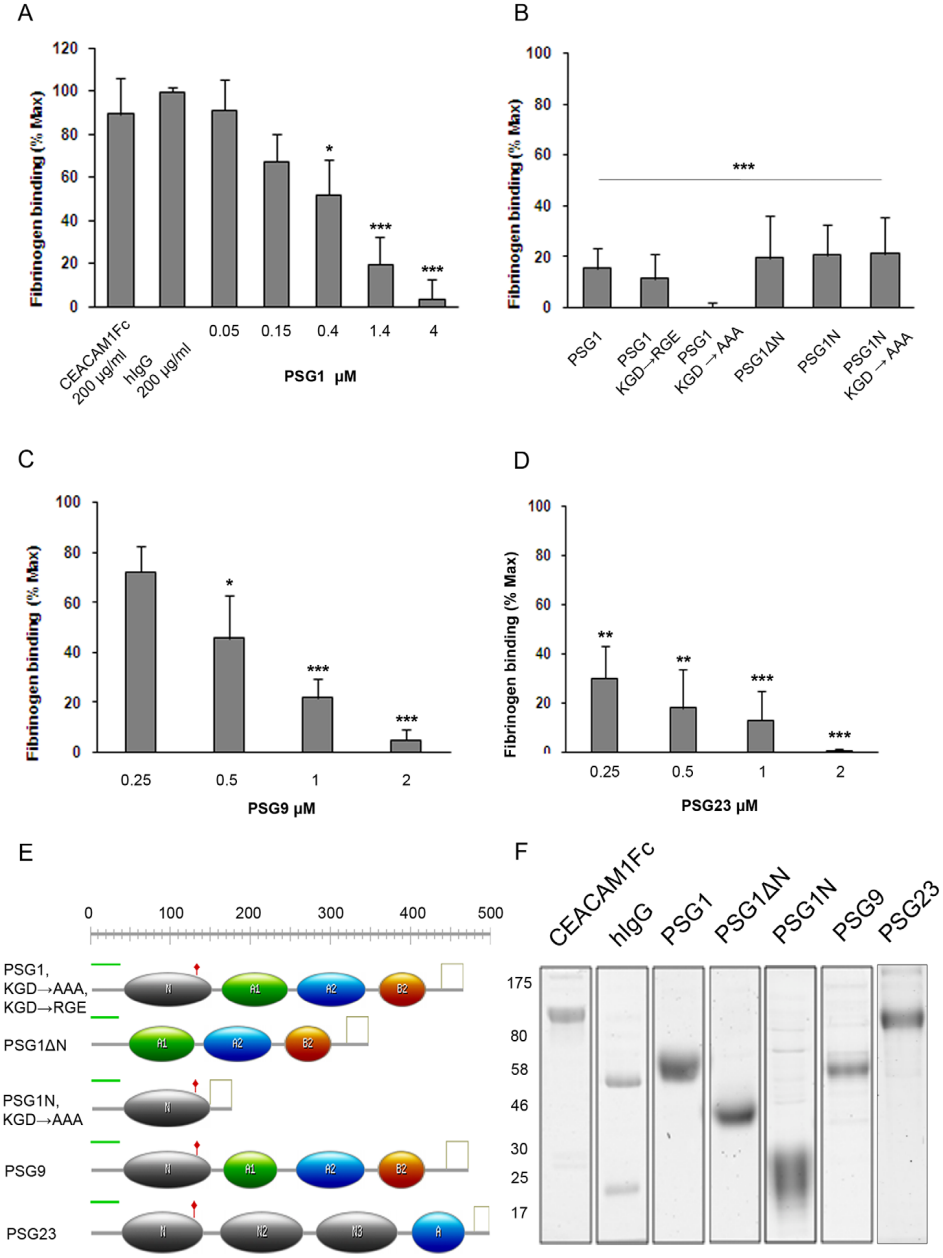
Human and mouse PSGs inhibit the platelet – fibrinogen interaction. PSG-mediated inhibition of the platelet – fibrinogen interaction was measured by estimating binding of Oregon Green-conjugated fibrinogen (OgFg) to washed human platelets using FACS. Fibrinogen binding to TRAP-activated platelets is set at 100% and resting platelets at 0%. All assays were analysed over a four or five point dose range of PSG proteins and mutants, from ∼5–100 or 200 µg/ml, depending on protein molecular weight. For clarity, some results are reported as single dose molar concentration comparisons between proteins. Protein molecular weights were calculated from amino acid sequences with no adjustments for posttranslational modifications. **a,** Binding of OgFg to human platelets in the presence of human CEACAM1, human IgG, and increasing doses of recombinant wildtype human PSG1. 4 µM PSG1 is equivalent to 200 µg/ml protein. **b,** Binding of OgFg to human platelets in the presence of (left to right): wildtype PSG1 (KGD); PSG1 in which the KGD tri-peptide motif is replaced with RGE, or AAA; PSG1 with deletion of N-domain; PSG1 N-domain; PSG1 N-domain in which the KGD tri-peptide motif is replaced with AAA. All proteins were used at 2 µM concentration, equivalent to 100 µg/ml full-length PSG1 variants, 75 µg/ml for PSG1ΔN, and 38 µg/ml for PSG1N variants. **c** & **d,** Binding of OgFg to human platelets in the presence of increasing concentrations of recombinant human PSG9 and mouse Psg23, respectively. 2 µM PSG9 and 2 µM Psg23 is equivalent to 100 µg/ml and 110 µg/ml, respectively. **e,** Summary of domain structures and mutants of PSG proteins used (see Fig. S3 in File S1 for sequences). **f,** Representative Coomassie-stained gels of protein used. For a - d, data are means of between three and seven independent experiments (detailed in main text) ± S.E.M. *, P<0.05; **, P<0.01; ***, P<0.001, nonparametric ANOVA with Dunnett’s multiple comparison post test.

PSG1 is the only PSG with a KGD, rather than RGD, motif on the exposed F-G loop of the protein N-domain, a motif with selective activity towards platelet integrin αIIbβ3 in the barbourin disintegrin [25]. We tested whether it exhibits anti-thrombotic activity by determining whether recombinant PSG1 made in HEK293T cells inhibits binding of an Oregon Green conjugate of human fibrinogen to Thrombin Receptor-Activating Peptide (TRAP)-activated washed human platelets. Fibrinogen binding was inhibited in a dose-dependent manner with >90% inhibition at physiological doses of PSG1 (200 µg/ml; n = 7). No inhibition was observed using 200 µg/ml IgG purified from human blood, or using 200 µg/ml of the PSG1-related CEACAM1 protein produced in HEK293 cells (Fig. 1a). Similar results were obtained using platelets activated with 25 µM epinephrine, 250 nM thromboxane mimetic U46619, and 10 µM adenosine diphosphate (ADP) (Fig. S2 in File S1), consistent with PSG1 inhibiting a pathway common to all four activation reagents tested. To determine whether inhibition was mediated by the KGD motif, we replaced KGD with RGE, a motif found on the homologous F-G loop of several mouse Psg protein N1-domains and routinely used as a non-functional analogue of the integrin-binding RGD motif; and with AAA, which is expected to abolish putative KGD-mediated integrin-binding of the F-G loop (Fig. 1e). Both mutants showed similar inhibitory activity to wildtype PSG1 indicating that the KGD is not essential for PSG1-mediated inhibition of the platelet – fibrinogen interaction (Fig. 1b; n = 3 for each mutant).

We therefore tested a mutant with a deletion of the entire PSG1 N-domain (PSG1ΔN) and found that it retained similar inhibitory activity in the platelet – fibrinogen binding assay to wildtype PSG1 (n = 4) (Figs. 1b, e; Fig. S3 in File S1). None of an extensive series of negative control proteins comprising nickel chromatography column eluates of culture medium from HEK293 cells sham-tranfected with empty pTT3 expression vector, high molar doses of V5-His tag peptide, PSG1-related CEACAM1 protein, and irrelevant proteins (rabbit and human IgG) showed inhibitory activity (Fig. 1a; Fig. S4 in File S1). Furthermore, we confirmed by protein sequencing that the N-terminal 35 amino acid predicted leader peptide, common to all our PSG proteins, was not present in the purified recombinant full length PSG1 protein (data not shown). These results indicate that more than one domain of PSG1 has anti-thrombotic activity. Therefore, to conclusively determine whether the N-domain has anti-thrombotic activity and whether this depends on the KGD motif, we made two proteins consisting solely of the PSG1 N-domain, one of which contained the intact KGD (N-KGD), while the other contained the substitution KGD → AAA (N-AAA). Both proteins showed inhibition of platelet – fibrinogen binding (n = 3), indicating that anti-thrombotic activity does indeed reside in the N-domain, in addition to at least one other domain of PSG1, and that the KGD motif is not critical for N-domain anti-thrombotic function (Figs. 1b, e). We were unable to specify the other domain(s) of PSG1 that have anti-thrombotic activity because interpretation of experiments using further domain deletion mutants (PSG1ΔA1, PSG1ΔA2, PSG1ΔA1A2, and PSG1ΔB2) was confounded by possible functional redundancy with the N domain. Also, it was not possible to produce PSG1 A1, A2, and B2 single domain proteins in sufficient quantities for our assays (data not shown; see Fig. S3 in File S1 for sequences). Representative Coomassie-stained gels of key protein preparations used in our assays are shown (Fig. 1f).

Immunomodulatory and proangiogenic functions of human and mouse PSGs are conserved, although the protein structural basis of these shared functions is unknown [9], [13], [16], [17]. Therefore we asked whether there is similar conservation of anti-thrombotic activity in human and mouse PSG families. We produced human PSG9 and mouse Psg23 proteins in HEK293T cells and tested them in the platelet – fibrinogen interaction assay. (See Fig. 1e and Fig. S3 in File S1 for PSG9 and Psg23 protein domain structures and sequences). Both proteins were inhibitory to a similar extent and over a similar dose range to PSG1 indicating conservation of anti-thrombotic function (Fig. 1c, d).

**Figure 2:**
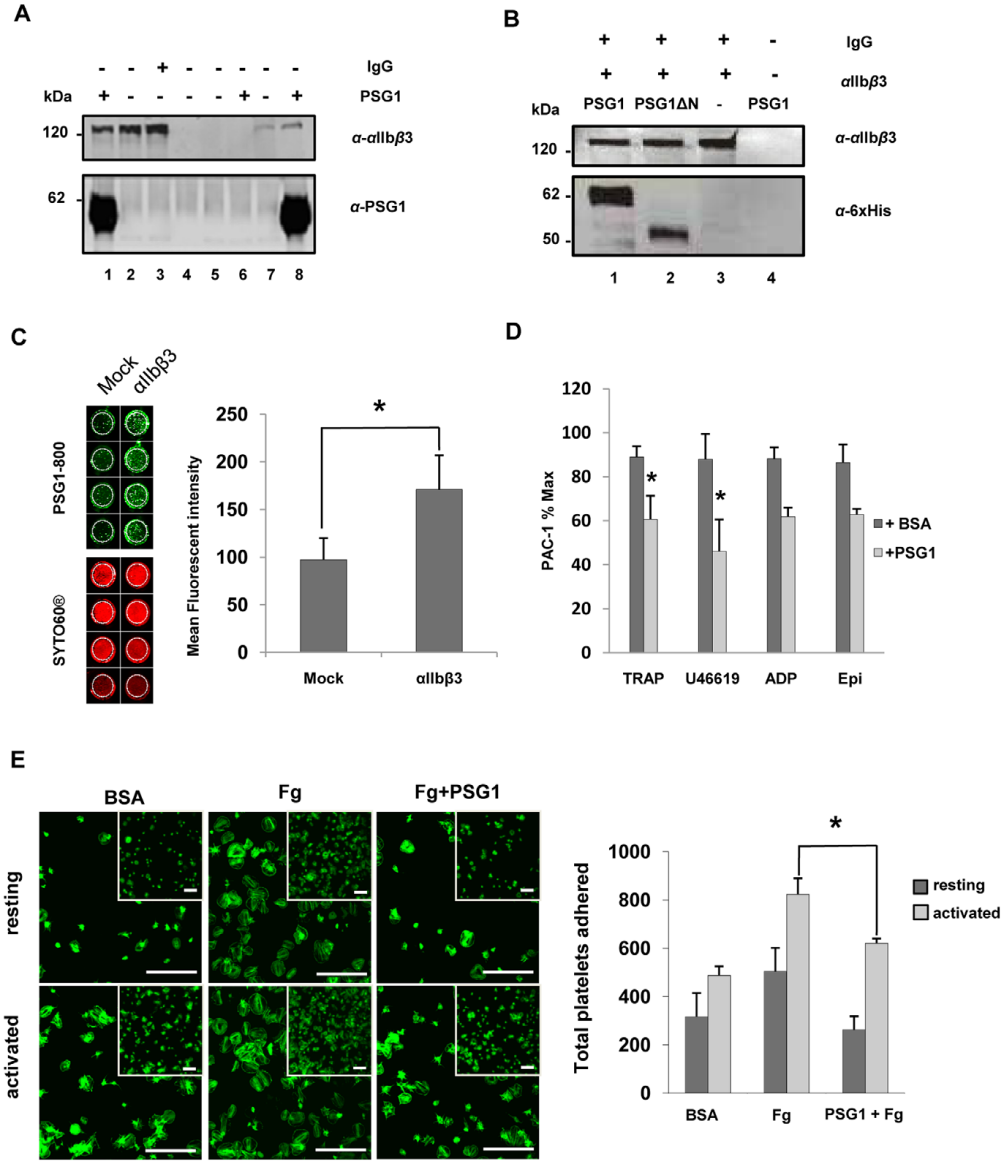
Multiple domains of human PSG1 bind the platelet integrin αIIbβ3. **a,** Integrin αIIbβ3 (2µg purified protein; lanes 1–3) pulls down PSG1 in an *in vitro* binding assay (lane 1). Negative controls are Protein G agarose beads with (lane 2) and without (lane 4) αIIbβ3, and with rabbit IgG instead of PSG1 (lane 3). Similarly, αIIbβ3 from lysates of CHO cell line stably transfected with αIIbβ3 (lanes 7, 8), but not lysate of sham transfected CHO control cell line (lanes 5, 6) pulls down PSG1 in co-immunoprecipitation assays. Negative controls lack PSG1, but contain α-αIIbβ3 mAb bound to beads (lanes 5 & 7). Western blotted membranes were probed with α-αIIbβ3 mAb Sz22 (upper gel) and α-PSG1 mAb-5 (lower gel). **b,** Commercial purified integrin αIIbβ3 bound to Protein G agarose beads pulls down recombinant PSG1 (lane 1) and PSG1ΔN (lane 2). Negative controls lack PSG1 (lane 3) or αIIbβ3 (lane 4). Western blotted membranes were probed with α-αIIbβ3 mAb Sz22 (upper gel), and α-His-Tag pAb (lower gel) which detects tagged PSG1 and PSG1ΔN proteins. **c**, Representative image and pooled data of fluorescent PSG1 (PSG1–800) binding to CHO cell line stably transfected with αIIbβ3 compared to sham transfected CHO control cell line. Cell density was measured using SYTO60. Data are means of six independent experiments ± S.E.M. *, P<0.05, Paired Student’s t-test. **d,** Binding of the activation-dependent monoclonal antibody, PAC-1, to platelet αIIbβ3. Washed human platelets were preincubated with BSA or PSG1 at 200 µg/ml before the addition of PAC-1 antibody and the indicated platelet agonist: TRAP (4 µM), thromboxane mimetic U46619 (250 nM), ADP (10 µM) or epinephrine (25 µM). Data are means of four independent experiments ± S.E.M. *, P<0.05, Student’s t-test. **e,** Washed platelets adhere and spread extensively on fibrinogen-coated (20 µg/ml) glass slides but poorly on 1% BSA-coated slides. Pre-incubation of platelets with 200 µg/ml PSG1 significantly reduced platelet adhesion and spreading on fibrinogen. Permeabilized platelets were stained for polymerized F-actin with Alexa-488 fluorescein isothiocyanate phalloidin before visualisation using confocal microscopy. Representative images are shown. Scale bar is 20 µm. Graph shows quantification of platelet adhesion as described in Methods. Data are means of three independent experiments ± S.E.M. *, P<0.05, Student’s t-test.

The dispensability of the KGD motif led us to question whether anti-thrombotic activity might be mediated by a mechanism other than antagonism of αIIbβ3 integrin, for example, by direct binding of PSG1 to fibrinogen. To rule this out, we tactically changed from fibrinogen competition assays and focused on the specificity of the PSG1– integrin interaction using Co-IP. We showed that PSG1 was pulled down by αIIbβ3 integrin from a purified preparation and from a CHO cell line stably transfected with human αIIbβ3 (Fig. 2a). No signal was obtained following pull-down using an empty vector-transfected control CHO cell line. In a further co-IP experiment, both wildtype PSG1 and PSG1ΔN were pulled down by purified αIIbβ3 supporting the existence of anti-αIIbβ3 activity in one or more of the A1, A2 or B2 domains (Fig. 2b). In similar experiments, we showed that recombinant full-length PSG1 and the PSG1 N-domain protein, with and without the KGD → AAA mutation, were pulled down by purified αIIbβ3 integrin, indicating that the KGD motif is dispensable for integrin binding by the PSG1 N-domain (Fig. S5 in File S1).

To provide additional independent evidence that PSG1– platelet interactions are mediated through binding integrin αIIbβ3, we showed that fluorescently-labelled PSG1 protein exhibited significantly increased binding to a CHO cell line stably transfected with human αIIbβ3 compared to an empty vector transfected CHO cell line (Fig. 2c). In addition, pre-treatment of platelets with PSG1 protein inhibited binding of PAC-1, a monoclonal antibody that binds only the activated form of integrin αIIbβ3 (Fig. 2d). Further evidence of direct binding of PSG1 to platelets, but not to fibrinogen, was produced by demonstrating that pre-incubation of both resting and activated platelets with soluble PSG1 at 200 µg/ml prevented their attachment on fibrinogen-coated glass slides, suggesting that PSG1 and fibrinogen compete for the same binding sites on platelets i.e. αIIbβ3 (Fig. 2e).

**Figure 3:**
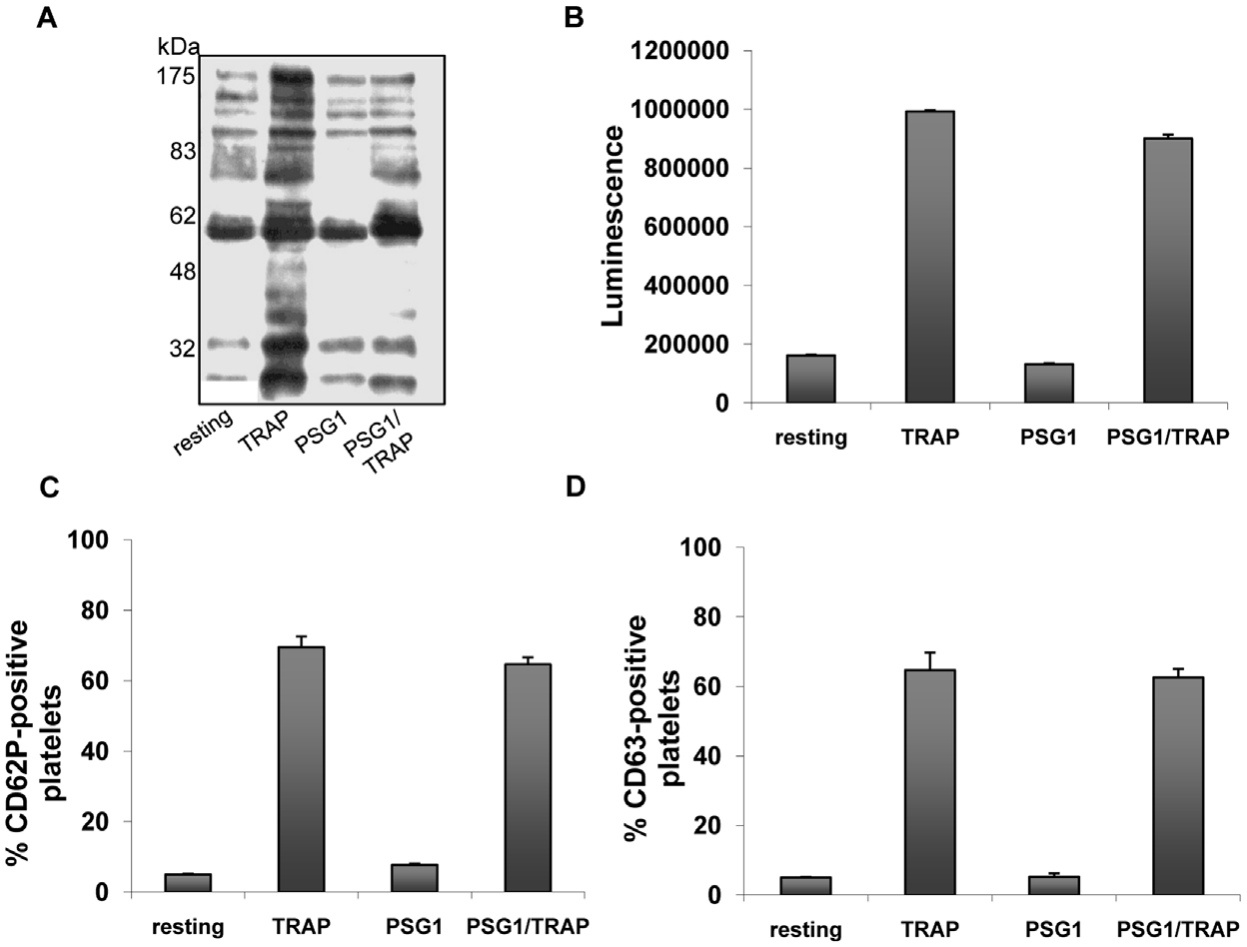
PSG1 does not activate platelets. **a,** Washed human platelets were treated at 37°C for 3 min with TRAP (4 µM) and/or PSG1 (200 µg/ml) for 2 min as indicated. Alternatively platelets remained untreated (resting). Platelet activation was assessed by analysis of the phosphotyrosine profile by western blotting with the antiphosphotyrosine mAb 4G10. Experiment was performed twice. **b, c** & **d,** In a similar series of experiments, three different markers of platelet degranulation were assessed. For ADP secretion assay (b), platelets were treated with 4 µM TRAP and/or PSG1 (100 µg/ml) for 3 min at 37^ O^C. For surface expression of CD62P (c) and CD63 (d) platelets were treated with 4 µM TRAP and/or PSG1 (100 µg/ml) for 10 min at RT as described in Methods. Alternatively platelets remained untreated (resting). Data represent the means of three independent experiments ± S.E.M.

We assessed whether PSG1 inhibits integrin function in platelets comparable to previously studied αIIbβ3 inhibitors [25]. We tested this by exposing washed platelets to 200 µg/ml PSG1 for 2 minutes at 37°C and measuring phosphotyrosine profiles on western blots of lysed platelets using anti-phosphotyrosine antibody (n = 2). PSG1 treatment did not increase protein phosphorylation compared to resting control treatment (Fig. 3a). However, pre-treatment of platelets with 200 µg/ml PSG1 for 2 minutes prior to TRAP activation reduced phosphorylation band intensity suggesting that PSG1 functionally inhibits platelet activation (Fig. 3a). This is consistent with our observations of PSG1-inhibited fibrinogen binding since phosphorylation events parallel integrin occupancy in platelets [29]. The integrin-specific nature of the PSG1-inhibition is also illustrated by the inability of PSG1 to affect other aspects of platelet activation. Activated platelets undergo degranulation causing release of ADP from dense granules, and increased surface expression of P-Selectin (CD62P) from alpha granules and CD63 from both lysosomes and dense granules [30]. We compared platelet ADP release and surface expression of CD62P and CD63 in TRAP activated platelets in the presence and absence of 100 µg/ml PSG1 (n = 3). Compared to robust responses to TRAP alone, PSG1 failed to affect any secretion responses demonstrating that the inhibitory effects of PSG1 on platelets are integrin αIIbβ3-specific (Fig. 3b, c, d).

**Figure 4:**
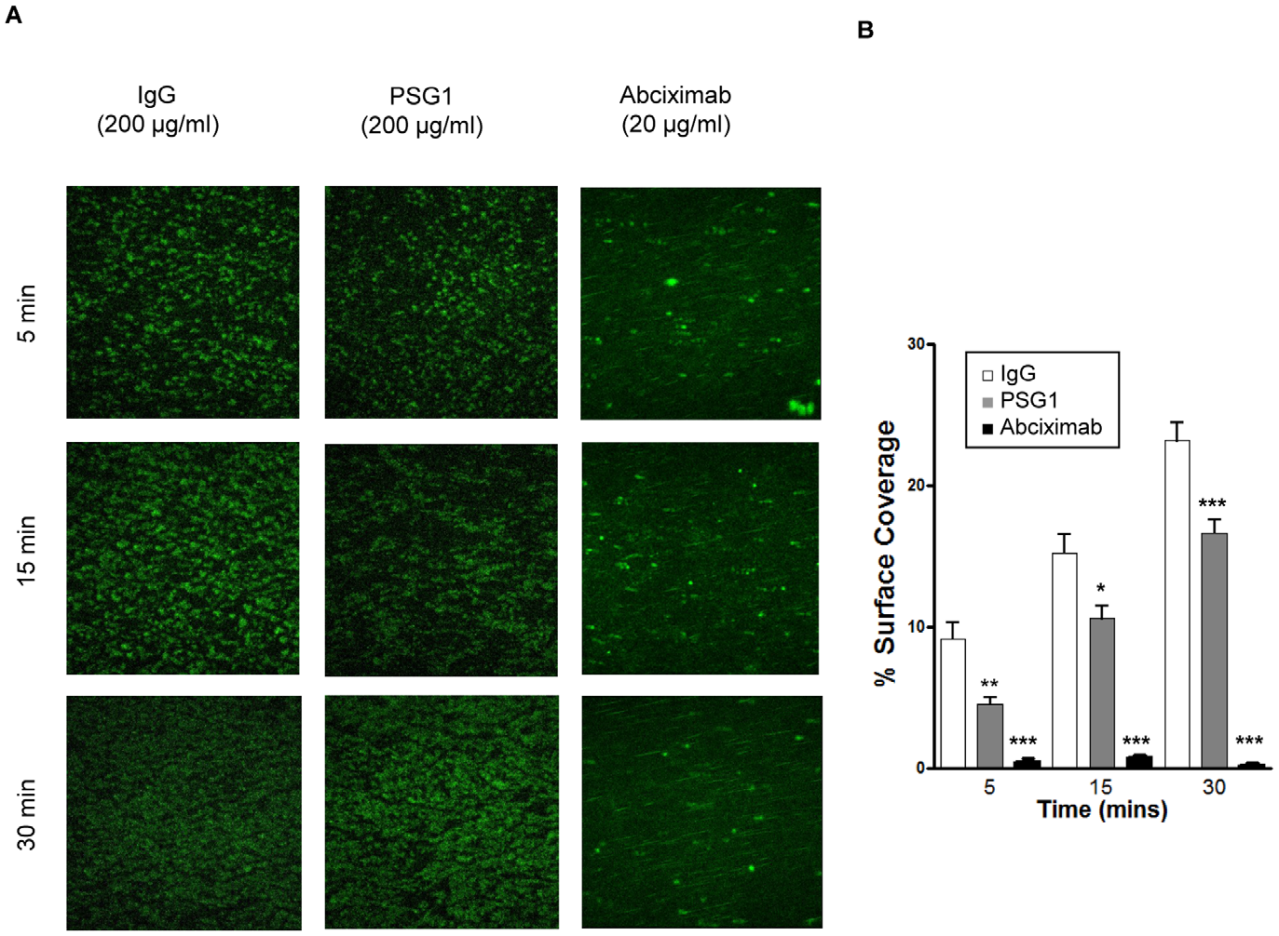
PSG1 is anti-thrombotic under arterial flow. **a & b,** Representative images of adhesion of platelets under arterial flow following addition of 200 µg/ml PSG1 to 3 ml of circulating whole human blood. 200 µg/ml rabbit IgG was used as a negative control. Abciximab, an αIIbβ3 antagonist, was used as a positive control. **b,** Summary data of eight replicated independent arterial flow experiments expressed as means ± S.E.M. *, P<0.05; **, P<0.005; ***, P<0.0005 vs IgG, Mann Whitney test.

We next determined whether PSG1 exhibits anti-thrombotic activity in a model of vascular flow using an experimental system in which human whole blood flows through a type 1 collagen-coated channel. We used a force of 15 dyn/cm^2^ which mimics arterial shear [56]. In operator-blinded experiments, 200 µg/ml PSG1 or 200 µg/ml rabbit IgG was added to blood and the extent of platelet adhesion was estimated by fluorescence microscopy at 5, 15 and 30 minutes after commencement of flow (n = 8). The αIIbβ3 antagonist Abciximab at 20 µg/ml was used as a positive control and, as expected, completely inhibited platelet deposition (n = 5). PSG1 reduced thrombus coverage at all time points compared to the IgG control, with a maximum reduction of 50% at 5 minutes (Fig. 4).

## Discussion

We show that human PSG1 and PSG9 and mouse Psg23 proteins inhibit the platelet – fibrinogen interaction suggesting that this may be a conserved function of all PSG proteins during pregnancy. This view is consistent with the fact that PSG gene families have evolved exclusively in mammals with haemochorial placentation, in which the fetal trophoblast is in direct contact with maternal blood (4).

Normal human pregnancy is associated with a nearly five-fold increased risk of venous thromboembolism, and pregnancy complicated by pathology is frequently associated with a hypercoagulable state in the maternal circulation [31], [32]. Normal pregnancy exhibits increased maternal expression of most procoagulant factors and reduced expression or activity of most anticoagulants [33]. The fetal trophoblast, which is in direct contact with maternal blood, actively regulates haemostasis by secreting procoagulant tissue factor (TF) and expressing anti-coagulant thrombomodulin (TM), endothelial protein C receptor (EPCR), and tissue factor pathway inhibitor (TFPI) [34], [35], [36]. The importance of local regulation of haemostasis at the maternal-fetal interface is supported by work in mouse models; for example, a mouse mutant lacking EPCR expression in the trophoblast giant cells exhibits thrombosis in the vascular bed and early fetal loss [37].

There is evidence that maternal platelets are activated in normal pregnancy potentially contributing to a hypercoagulable state [38], [39], and suggesting the existence of counteracting physiological mechanisms that prevent platelet aggregation and thrombosis. This is consistent with the anti-thrombotic role for PSGs suggested by our findings, and with the reduction in maternal blood PSG levels observed in pro-thrombotic disorders of pregnancy [20], [21]. Preeclampsia is associated with increased activation of clotting pathways, platelet activation and increased platelet consumption due to hypercoagulability [40]–[45], and the increased release of inflammatory mediators from activated platelets may also contribute to endothelial dysfunction [46]. Therefore, we can speculate that reduced PSG expression may contribute to the maternal syndrome in preeclampsia by facilitating fibrinogen binding to activated platelets. Interestingly, in a LC-MS/MS proteomics screen of the SCOPE collection of preeclampsia maternal blood samples taken at 20 weeks, increased fibrinogen gamma chain expression provided one of two strong predictors of subsequent development of preeclampsia [47].

Although the idea that PSGs might function analogously to snake venom disintegrins has been current for some time [24], experimental evidence has been lacking. Our analysis of the PSG1 interaction with the platelet integrin αIIbβ3 was initially prompted by the presence of a KGD tri-peptide motif on a solvent-exposed loop of the protein N-domain. Uniquely among snake venom disintegrins, the barbourin protein found in the venom of the pygmy rattlesnake has a KGD tripeptide motif that mediates specific binding to integrin αIIbβ3 [25]. However, our mutagenesis analysis of the PSG1 N-domain indicates that the KGD motif is not required for binding of the PSG1 N-domain to αIIbβ3, or for its anti-platelet function. Moreover, both human PSG9 and mouse Psg23, neither of which contain a KGD motif, also have anti-platelet activity further suggesting that a KGD is not critical. Deletion of the entire PSG1 N-domain (PSG1ΔN) also had no effect on the ability of the residual A1–A2–B2 domain protein to inhibit fibrinogen binding. Therefore, the PSG1 protein contains multiple domains that can independently inhibit the platelet - fibrinogen interaction. We were unable to specify which or how many of the other PSG1 domains (A1, A2, B2) are responsible for this activity because we could not produce the A1, A2, and B2 single domain proteins in sufficient quantities and at acceptable purity for our assays. Nevertheless, we can conclude that the N-domain and at least one other PSG1 domain can interact with platelets and inhibit fibrinogen binding. We speculate that the existence of multiple PSG1 protein domains mediating its anti-platelet function may provide favourable binding kinetics to the platelet integrin; however, investigation of this hypothesis was beyond the scope of the current study.

The proposal that PSGs may act similarly to disintegrins is supported by our finding that treatment with PSG1 does not cause platelet activation as determined by a variety of assays including phosphotyrosine blots and degranulation assays. Therefore, we conclude that the sole function of PSG binding to αIIbβ3 is to inhibit fibrinogen binding and prevent aggregation of activated platelets in the context of normal pregnancy, and not to engage in outside-in signalling. This novel function is distinct from the observed PSG-mediated induction of Th2 cytokine release from monocytic, endothelial and trophoblastic cell lineages which may be mediated by binding cell surface receptors such as CD9 and surface proteoglycans [13], [18], [22], [23]. Therefore, PSGs may have a dual role in pregnancy: modulation of the cytokine milieu underpinning immunomodulatory and angiogenic functions, and anti-platelet activity which may underpin an anti-thrombotic function in the placental bed or in the maternal circulation. Indeed, given the intimate regulatory links between the innate immune system and the blood coagulation systems in pregnancy [48], the hitherto described immunoregulatory functions of PSGs may be as relevant to their anti-thrombotic function proposed herein as to the currently accepted paradigm of protection of the allogeneic fetus from the maternal immune system.

The high levels of PSGs reported in maternal blood at term is difficult to explain as due to an immunoregulatory function because a PSG1 level as low as 2.5 ug/ml can induce functionally significant levels of TGFβ1 [14]. We suggest that the requirement to compete for αIIbβ3 binding on activated platelets with abundant (3 mg/ml) fibrinogen present in the maternal bloodstream may explain the high level of PSG expression. We note that at their observed concentrations, PSGs and fibrinogen would be approximately equimolar in the maternal blood. However, an alternative proposal, based on the observation that many placental hormones are encoded by multigene families, is that selection for increased gene dosage is the outcome of co-evolutionary arms races underpinned by maternal - fetal conflict [49]. However, it is unclear why regulation of platelet aggregation in the context of normal pregnancy would be a substrate for maternal – fetal conflict because deregulation of haemostasis would likely have adverse consequences for both mother and fetus.

Within the scope of this study, we were unable to purify sufficient quantities of endogenous human PSG1 protein from maternal blood to provide direct evidence of an anti-platelet function. However, in our assays, we carried out a series of protein controls comprising rabbit and human IgG purified from blood, and recombinant CEACAM1 made in HEK293 cells. None of these control proteins exhibited anti-platelet activity in our assays, whereas all tested PSG protein variants did. Since PSG1 and CEACAM1 are closely related members of the immunoglobulin superfamily, we conclude that PSG1 mediated inhibition of fibrinogen binding to platelets is specific and accurately reflects the function of the endogenous protein in vivo. In summary, we propose that PSGs, which are found exclusively in species with haemochorial placentation, may have evolved to prevent platelet aggregation and thrombosis at the placental surface or in the maternal circulation in the prothrombotic environment of pregnancy. A corollary is that the necessity to antagonise abundant (3 mg/ml) circulating fibrinogen may explain the high level of PSG expression during human pregnancy.

## Materials and Methods

### Ethics Statement

Human platelets used in this study were obtained from healthy volunteers that were free of medication. Patients were provided with a detailed information sheet and gave written consent under RCSI Ethical Review Protocol approved by the RCSI Research Ethics Committee.

### Production of Recombinant PSG Proteins and PSG1 Mutant Proteins

As described [26], the PSG1a (Ensembl PSG1-001) open reading frame (ORF) was amplified by polymerase chain reaction (PCR) from an existing cDNA clone [50] incorporating restriction sites (*Xho*I at the 5′ end and *EcoR*I at the 3′ end) to allow insertion into the pBlueBac4.5V5/His vector (Invitrogen, UK). Both the amplified PCR product and the vector were digested with the above restriction enzymes and separated by gel electrophoresis on a 1% agarose gel. The relevant bands were cut from the gel and the DNA extracted using a Qiagen Gel Extraction kit (Qiagen, UK), quantified by UV spectrophotometry and gel electrophoresis, then ligated using T4 DNA Ligase (New England Biolabs (NEB), USA). The ligation reactions were then transformed into TOP10 chemically competent E.Coli (Invitrogen, UK) and plated onto Luria Bertani (LB) agar plates, containing ampicillin (100 µg/ml) for selection, and grown overnight at 37 ^o^C. Colonies were picked and grown overnight in LB broth containing ampicillin (100 µg/ml). The plasmid DNA was then extracted from the bacterial cultures using a Qiagen Mini-Prep kit (Qiagen, UK) and analysed by diagnostic restriction digest to confirm presence of an insert. Clones were sent for sequencing (Dept of Biochemistry, Oxford University, UK). A positive clone was selected, the PSG1pBlueBac4.5V5/His bacterial culture was expanded further and the DNA purified using a Qiagen Midi-Prep kit (Qiagen, UK).

The PSG1 ORF, including the carboxyl terminal V5/His tag, was subsequently re-amplified by PCR, incorporating *EcoR*I sites at the 5′ and 3′ ends. Both the resulting PCR product and pTT3 expression vector [51] were digested with *EcoR*I and cloning was carried out as described above.

To produce recombinant PSG1 protein, endotoxin-free plasmid DNA was purified from PSG1pTT3 bacterial cultures using the Endofree Plasmid Maxi Kit (Qiagen, UK). All subsequent steps were carried out using confirmed endotoxin-free reagents and tissue culture flasks. The DNA was transiently transfected into Freestyle 293-F cells (Invitrogen, UK) using Freestyle MAX reagent (Invitrogen, UK). The Freestyle 293-F cells were grown in suspension in Freestyle 293 Expression Medium, by shaker culture, to a density of 1×10^6^ cells per ml. The plasmid DNA was diluted in OptiPRO Serum Free Medium at a ratio of 1 µg DNA in 20 µl OptiPRO for every 1 ml of cells. Freestyle MAX reagent was also diluted in OptiPRO at the same ratio (1 µl Freestyle MAX reagent in 20 µl OptiPRO per ml of cells). The diluted DNA and Freestyle MAX reagent were then combined, mixed gently and incubated at room temperature (RT) for 20 minutes (min). The mixture was added to the cell suspension and the cells were cultured for a further 72 hours (h). The culture was then centrifuged at 1,000 rpm for 5 min at RT to separate the protein-containing medium from the cells, and the medium was frozen in aliquots at minus 80 ^o^C.

Recombinant PSG1 protein was purified from cell culture medium using Qiagen Ni-NTA resin. Imidazole (Sigma, UK) was added to the culture medium to a final concentration of 10 mM to reduce non-specific binding. Ni-NTA resin was added to the medium at a ratio of 1 ml resin suspension (corresponding to 0.5 ml resin bed volume) to 100 ml medium. The medium and resin were then batch bound overnight on a rotating wheel at 4 ^o^C. The medium and resin mix was then passed through a disposable polypropylene column (Pierce, Thermo Fisher Scientific, Ireland) and the resin was washed with wash buffer (500 mM NaCl, 20 mM NaH_2_PO_4_, pH6) until the absorbance at 260 nm reduced to 0. Protein was then eluted from the column with increasing concentrations of imidazole in wash buffer, usually 4×1.5 ml 50 mM fractions, 5×1.5 ml 200 mM fractions, 4×1.5 ml 300 mM fractions and 3×1.5 ml 500 mM fractions. PSG1 protein was generally observed to elute in the last 50 mM fraction, the five 200 mM fractions and the first 300 mM fraction. These PSG1 containing fractions were then pooled and passed through a new column containing 0.5 ml Ni-NTA resin, the flow-through collected, and bound protein eluted with 1 ml 50 mM Imidazole followed by three 1 ml 200 mM Imidazole fractions. The flow-through and three 200 mM fractions were pooled and concentrated to a volume of 4 - 6 ml using a Millipore Amicon Ultra Ultracel 10K centrifugal filter (Millipore, Ireland). The concentrate was then dialysed against three changes of 2 L of phosphate buffered saline (PBS) at 4 ^o^C. The protein was then further concentrated to a volume of 1 - 2 ml depending on the starting volume of culture medium. Protein was quantified by Bradford Assay or UV Spectroscopy, checked by polyacrylamide gel electrophoresis, tested for LPS contamination (Limulus Amebocyte Lysate QCL-1000; Cambrex BioScience, Germany) aliquoted and frozen at minus 80 ^o^C.

Variants of the PSG1 protein ORF were created using site-directed mutagenesis of the PSG1pTT3 plasmid and the modified plasmids were used to produce PSG1 protein variants as described for PSG1pTT3 above (sequences of all variants are listed in Fig. S3 in File S1). Primers were designed to allow amplification by PCR of the required PSG1 domains in each instance, thereby deleting undesired PSG1 sequences from the vector. PCR was carried out using Finnzymes Phusion Hot Start DNA polymerase (Finnzymes, NEB, UK), the resulting PCR product was checked by gel electrophoresis and then ligated using T4 DNA Ligase (NEB, UK). Ligation reactions were transformed into NEB Turbo Competent cells and plated on LB agar plates containing ampicillin (100 µg/ml) and grown overnight at 37 ^o^C. Colonies were picked and grown overnight in LB broth containing ampicillin (100 µg/ml) at 37°C with shaking. Plasmid DNA was then extracted from the overnight cultures using a Qiagen spin mini-prep kit (Qiagen, UK) and the plasmid DNA was sent for sequencing (GATC-Biotech, Konstanz, Germany). Selected clones were used for mutant PSG1 protein production as described above for the wildtype PSG1 protein.

### Preparation of Human Platelets

Platelets were collected into 0.15 vol/vol acid-citrate dextrose (ACD, 75 mM trisodium citrate, 124 mM dextrose and 38 mM citric acid) anticoagulant and washed using a modification of a previously described method [52]. Briefly, blood was centrifuged at 150 × g for 10 min at RT. In order to avoid any contamination from the buffy coat, ∼0.5 ml of the platelet-rich suspension above the buffy coat layer was left behind in the centrifugation tube. Platelet-rich plasma (PRP) was then acidified to pH6.5 with ACD and Prostaglandin E_1_ (PGE_1,_ 1 mM) was added. The platelets were pelleted by centrifugation at 750 g for 10 min at RT. The supernatant was removed and the platelet pellet was gently resuspended in 130 mM NaCl, 3 mM KCl, 10 mM trisodium citrate, 9 mM NaHCO3, 6 mM dextrose, 0.9 mM MgCl2, 0.81 mM KH2PO4, 10 mM Tris pH7.4 (JNL buffer). Platelet count was adjusted to 3×10^8 ^per ml using a ***Sysmex*** XE K-1000 counter (Toa Medical Electronics Co. Ltd, Kobe, Japan). Platelets were allowed to stand at RT for 45 min to let PGE_1_ dissipate. Calcium chloride (CaCl_2_, 1.8 mM) was added to platelets immediately before use.

### Platelet Aggregation Assay

Platelet aggregation was performed at 37°C in a BioData Corporation PAP-8 aggregometer (Horsham, PA, USA). 250 µl of washed platelets were used per assay and were incubated at 37°C in JNL buffer with or without 200 µg/ml wildtype PSG1 protein. Aggregation was monitored for 5 min in the presence of either 4 µM TRAP, 250 nM U46619, 10 µM ADP, or 25µM epinephrine. Percentage of aggregation was determined by percentage of light transmission. A 100% aggregation baseline is set in the aggregometer by measuring the light transmission through the modified HEPES buffer [53].

### Fibrinogen Binding Assay

10 µl of 2.5 mg/ml Oregon Green 488 fibrinogen-conjugate Oregon green labeled Fibrinogen (OgFg; Invitrogen, UK) was added to 20 µl aliquots of washed platelet suspension along with the indicated concentrations of wildtype PSG1, PSG1 variant proteins, PSG9 or Psg23 in successive tubes. Dose - response curves were produced for all proteins using serial dilutions of either 1∶2 or 1∶3 in the range 100 or 200 µg/ml to ∼5 µg/ml. For clarity of presentation, comparisons between proteins of different molecular weights are expressed in micromolar concentrations (µM). Molecular weights of proteins were calculated based on amino acid sequence, with no account taken of post-translational modifications. All experiments were run in duplicate at least three times. The platelet suspension was vortexed and allowed to stand at RT for 10 min before the addition of 3.4 µM thrombin receptor activating peptide (TRAP, Bachem, UK), a dose known to produce a 50% maximal response as measured by aggregometery in pilot studies. Assay tubes were incubated at RT for a further 10 min. The reaction was stopped by addition of 1 ml ice-cold buffer. In a separate series of experiments, 250 nM U46619 (Tocris Bioscience, UK), 10 µM ADP (Bio/Data Corporation, UK), or 25 µM epinephrine (Bio/Data Corporation, UK) were used to activate platelets The association of OgFg with platelets was detected using a fluorescence-activated cell sorter (Becton Dickinson). Data acquisition and analysis were performed with the Cell Quest program. Platelet populations were gated, and histograms of mean fluorescence were generated for each sample. Statistical analysis was performed on the geometric scale.

Rabbit IgG (I5006-10 mg, Sigma, UK), Human IgG purified from human blood, PSG1-related protein CEACAM1-Fc produced in HEK-293 cells, and V5-Hisitidine (V5-His) peptide were used as negative controls. The V5-His tag peptide (N-GKPIPNPLLGLDSTRTAHHHHHH- C) was obtained from Eurogentec, UK. To rule out non-specific inhibition from the protein purification procedure, eluates from the Ni-NTA resin column that did not contain PSG, but contained other contaminant proteins, were run in parallel with PSG proteins. To confirm that the 35 amino acid leader sequence, which is common to all human PSG proteins analysed is cleaved as predicted, a purified preparation of PSG1 protein was N-terminal sequenced (Alta Bioscience, Birmingham, UK).

### PAC-1 Binding Assay

As described [53], washed platelet aliquots (10µl) were dispensed in 15 mm plastic tubes and pretreated with 200 µg protein (PSG1, control proteins or mutants) for 15 minutes in a final volume of 80 µl. 10 µl of FITC labeled PAC-1 antibody and agonist (10 µl, to final concentration of 4 µM TRAP, 250 nM U46619, 10 µM ADP and 25 µM epinephrine) were then added and the reaction was allowed to proceed for 10 min. The assay was terminated by addition of 1 ml ice-cold HEPES buffer. Platelet associated fluorescence was estimated by using a FACSCaliber flow cytometer. Platelet populations were gated, 10,000 events were counted, and histograms of mean fluorescence were generated for each sample. Statistical analysis was performed on the geometric scale. Human IgG protein and BSA were used as negative controls.

### Detection of CD62P and CD63

As described [54], Platelet alpha-granule release was monitored using a phycoerythrin (PE) conjugated α-P-selectin (α-CD62P) mAb (1∶10; BD Biosciences, UK). Alternatively, dense granule secretion was assessed using a FITC-conjugated α-CD63 mAb (1∶10; BD Biosciences, UK). Platelet aliquots (20 µl) were diluted 1∶1 in JNL buffer and either left untreated, treated with 4 µM TRAP, or treated with PSG1 (100 µg/ml) for 10 min at RT. α-CD62P or α-CD63 mAbs were added to the platelet suspension and incubated for 20 min at RT. Tubes were vortexed immediately after addition of antibodies and at the end of 20 min incubation. 1 ml ice cold JNL buffer was added to each tube to quench activation and samples were read immediately on FACS Calibur (BD Biosciences, UK). Platelet populations were gated, 10,000 events were counted and histograms of mean fluorescence were generated for each sample. The experiment was performed three times with duplicated samples. Data were expressed as the percentage of antibody-positive platelets.

### Platelet Dense-granule Secretion Assays

ADP-secretion from platelet dense granules was assessed as previously described [55]. Washed platelets were prepared as described above and 70µl aliquots of the platelet suspension (1.5×10^8 ^per ml) were added to the wells of an all-white 96-well microplate (Packard Instrument Company, USA) and incubated with TRAP and/or PSG1 (100 µg/ml) in a final volume of 100 µl at RT, after which the plate was placed on a Delfia plateshake (model 1296-004, PerkinElmer, USA) for 3 min at 37°C. 10 µl Chrono-lume (Chrono-Log, USA) were injected into each well and luminescence in arbitrary units was detected for 1 sec, following a 1 sec delay, using a Wallac 1420 Multilabel Counter (Perkin Elmer). The experiment was performed three times with duplicated samples. Data are expressed as luminescence (arbitrary units).

### Platelet Adhesion Assay

Washed platelets were prepared as above. Polysine slides (25×75×1 mm; Thermo Fisher Scientific, Ireland) were coated overnight with human fibrinogen (20 µg/ml; Calbiochem, UK). Control slides were coated with 1% BSA (w/v). All slides were blocked with 1% BSA for 2 hours at 37°C. Washed platelets (1.5×10^8 ^per ml) were added to slides and allowed to adhere for 45 min. For TRAP-activated platelet studies, TRAP (4 µM) was added immediately before adding platelets to the slide. For analysis of the effect of PSG1, protein (200 µg/ml) was pre-incubated with washed platelets for 10 min at RT prior to addition to fibrinogen coated slides as described above. After 45 min slides were fixed in 3.8% (v/v) paraformaldehyde and permeabilised with Triton X-100 (0.1% v/v), followed by blocking in 5% goat serum in TBS for 30 min at RT. Adhered platelets were probed for polymerised actin using Alexa Fluor 488-phalloidin (1∶300; Invitrogen, UK). Stained platelets were mounted using Dako fluorescent mounting medium. Images were acquired sequentially using a Zeiss LSM 710 Confocal microscope. Platelet adhesion was quantified as described [54].

### Phosphotyrosine Analysis using Western Blotting

Washed platelets were prepared as described above. Essentially as described [53], 450 µl of washed platelets (3×10^8 ^per ml) were stirred at 1,100 rpm at 37°C for 1 min before addition of 200 µg/ml PSG1 for 2 min, followed by addition of 4 µM TRAP for 3 min. Positive and negative controls were obtained by incubating platelets with or without 4 µM TRAP, respectively, in the absence of PSG proteins. Samples were solubilized in ice-cold 10X lysis buffer (50 mM ethylmaleimide, 10% Triton X-100, 5% N-octylglucoside, 10 mM sodium orthovanadate, 20 mM PMSF, 200 µg/ml soya bean trypsin inhibitor, 10 mM EDTA, 100 mM benzamidine, pH 7.4) for 1 h and stored at minus 80°C until needed. Platelet lysates were separated on 7.5% SDS polyacrylamide gels transferred onto a polyvinylidene fluoride (PVDF) membrane. After transfer, the PVDF membrane was blocked with 5% BSA/TBS for 2 h and washed with TBS-T buffer. The membrane was incubated with α–phosphotyrosine mAb (1∶1000; clone 4G10, Millipore, Ireland) overnight at 4°C followed by anti-mouse conjugated with horseradish peroxidase (HRP) (1∶20,000; Abcam, UK) for 1 h at RT. Immunoreactive bands were visualized using Immobilon Western HRP-substrate chemiluminescence detection kit (Millipore, Ireland) according to manufacturer’s instructions.

### Fluorescent PSG1 Binding Assay

1 mg of recombinant PSG1 was labelled with an *N*-hydroxysuccinimide (NHS) ester-activated Dylight®-800 fluorophore (Thermo Scientific, UK), as per the manufacturer’s instructions. Labelled protein was aliquoted and stored at −20°C. Integrin α_IIb_β_3_ dual-transfected CHO-K1 cells [53] were enriched for a highly expressing population using the FACS Aria II system (BD Biosciences, UK). Briefly, mock and integrin α_IIb_β_3_ dual-transfected CHO-K1 cells were non-enzymatically detached (0.2% EDTA) and washed in cell sorting buffer (1% FBS, 1 mM EDTA, 25 mM HEPES in PBS). 1×10^6^ cells in 100 µl of cell sorting buffer were incubated with 20 µl of Phycoerythrin-conjugated mouse anti-CD41a (BD Pharmingen, UK) for 30 minutes on ice in the dark. Cells were washed twice in 5 ml of cells sorting buffer, resuspended in 1 ml and sorted as indicated. Enriched cells were propagated and used in Fluorescent Ligand Binding Assay. Sorted integrin α_IIb_β_3_ dual-transfected CHO-K1 cells and mock-transfected controls were seeded in quadruplicate in 96-well plates in selective media at 60,000 cells per well and allowed to adhere overnight. The next day the wells were washed in PBS and 100 µl selective media with 50 µg/ml of labelled PSG1 was applied to the cells for 30 minutes at 37°C. Wells that had been blocked with media overnight were used as controls for background binding of the labelled PSG1. Media was removed and wells were washed extensively and fixed in fresh 4% paraformaldehyde-PBS pH 7.4 for 20 min at RT or overnight at 4°C. The cells were washed once with PBS and a 1 in 10,000 dilution of the SYTO®60 red fluorescent nucleic acid stain (Molecular Probes, UK) in dH_2_O was applied for 1 hr at RT. Wells were washed three times with dH_2_O and plate allowed to dry. The plate was scanned on a LI-COR Odyssey® Infrared Imaging System using the manufacturer’s microplate settings. The mean fluorescence intensity for each well in the red and green channels was determined using the supplied software. Data from the green channel were normalized to the red channel. Statistical analysis was performed using the Paired Student’s t-Test from the Graph Pad Prism 4 statistical analysis software package.

### Protein Pull-down Assays

To bind integrin αIIbβ3 to Protein G agarose beads, 2 µg of purified αIIbβ3 protein (Calbiochem, UK) and 3 µg mouse anti-αIIbβ3 mAb (clone SZ22, Beckman Coulter, Ireland) were added to a tube containing 700 µl of Buffer A (100 mM NaCl, 0.1% Triton-X 100, 1 mM CaCl_2_, 20 mM TrisHCl pH 7.4) overnight at 4°C with gentle rocking. Immune complexes were obtained by adding 40 µl of Protein G agarose beads (Amersham, UK) for 3 h at 4°C followed by three washes with ice-cold Lysis buffer consisting of Tris HCl pH 7.4, 150 mM NaCl, 1% NP40 plus the tyrosine phosphatase inhibitor Na_3_VO_4_ (1 mM) and the protease inhibitors PMSF (1 mM), pepstatin (1 µM) and aprotinin (1.5 µg/ml).

To obtain integrin αIIbβ3-containing lysates from cells, CHO cells that were mock transfected or stably transfected with platelet integrin αIIbβ3 (Ref. 53) were washed with PBS and lysed in Lysis buffer (see above). After incubation at 4°C for 20 min, nuclear and cellular debris were removed by microcentrifugation at 14,000 rpm for 15 min at 4°C. For immunoprecipitation of integrin αIIbβ3 from CHO cells by binding to Protein G agarose beads, extracts were initially pre-cleared using bovine serum albumen (BSA, Sigma, UK) coated protein G agarose beads (15 µl beads per 700 µg of total protein in 700 µl lysis buffer) by incubation at 4°C for 1 h with gentle rocking. The lysates were recovered from the beads by centrifugation at 3,000 rpm for 3 min and transferred to fresh tubes for incubation with 3 µg anti- αIIbβ3 mAb overnight at 4°C with gentle rocking. Immune complexes were obtained by adding 40 µl of Protein G agarose beads for 3 h at 4°C, followed by washing three times with ice-cold Lysis buffer.

For the *in vitro* binding assay, the last wash was removed from each of the samples described above and the Protein G agarose beads were incubated with PSG1 or PSG1ΔN. Rabbit IgG (I5006-10 mg, Sigma, UK) was used as a negative control. All samples were incubated on a rotator for 1 h at RT. The beads were recovered by centrifugation at 3,000 rpm for 3 min followed by extensive washing to remove unbound PSG proteins. Protein was removed from the beads by boiling for 5 min in 20 µl of 2X SDS-PAGE sample buffer before electrophoresis and western blot analysis.

For western blot analysis, protein samples were resolved by SDS-PAGE on 4 - 20% gradient gels and then transferred to nitrocellulose membranes, which were blocked for 1 h at RT in triphosphate buffered saline (TBS) containing 0.05% Tween 20 (TBS-T) and 5% milk (w/v). Mouse anti-PSG1 mAb-5 (D. Shanley et al., manuscript in preparation), anti- αIIbβ3 mAb and anti-His-Tag pAb (1∶1000 dilution; Abcam ab9108) primary antibody incubations were overnight at 4°C. Secondary antibody incubations were at RT for 1 h. In all cases we used Alexa Fluor 680 and 800-coupled anti-mouse secondary antibodies (LI-COR Biosciences, UK) for detection with the Odyssey® infrared imaging system (LI-COR Biosciences, UK).

### Thrombus Deposition under Arterial Shear

The effect of PSG1 on platelet thrombus coverage of collagen under arterial flow was examined using a modification of a previously described recirculating pump system (ibidi, Munich, Germany) [56]. Whole human blood was drawn into a 10 ml syringe containing 1 ml of sodium citrate anticoagulant resulting in a final concentration of 0.38% w/v of sodium citrate. The blood was fluorescently labelled by addition of 10 µl of a 10 mM aqueous stock solution of mepacrine (Sigma, UK) resulting in a final concentration of 10 µM, and incubated for 30 min at 37°C. Perfusion slides (µ-slide VI, ibidi, Munich, Germany) with chamber dimensions of 5 mm width, 30 mm length and 0.4 mm channel height were coated with fibrillar Type I Collagen (100 µg/ml; Helena Laboratories, UK), since this type of collagen promotes thrombus formation to a greater extent than acid solublised Type I collagen. Using the ibidi pump system with a 1.6 mm tubing diameter and 5 ml syringe configuration, and a 3 ml perfusate volume, labelled blood was perfused over collagen at arterial shear (15 dyn/cm^2^). The appropriate air pressure and flow rate was calculated using the ibidi pump control software V1.3 (ibidi, Munich, Germany) allowing for relative blood viscosity of 4. At each time point, five images were taken along the channel length using fluorescent microscopy (Nikon T2000E). The percentage of thrombus coverage was quantified using NIS Elements BR software (Version 3.0, Nikon, Japan).

## Supporting Information

File S1 (DOC)
