# Supplementary material for: Pregnancy-Specific Glycoproteins Bind Integrin αIIbβ3 and Inhibit the Platelet—Fibrinogen Interaction
Source: PLoS One. 2013 Feb 28;8(2):e57491. doi: 10.1371/journal.pone.0057491 (PMC3585349; doi:10.1371/journal.pone.0057491)
Supplement: File S1 — (DOC) [file pone.0057491.s001.doc]

**Supplementary File 1**

**Figure S1. PSG1 mRNA is relatively highly expressed compared to other PSGs in first trimester and term placenta.**

**Figure S1 Legend.** Four first trimester and four term placentas (2 male and 2 female at each stage) were collected and cDNA was prepared as described below. Three sets of redundant PCR primers - two sets spanning the first intron (I1a, I1b) and one within the fifth exon (E5) - were used to amplify PSG transcript cDNAs. PCR products were cloned and sequenced to quantify relative expression of individual PSGs. Data are plotted for each a, placenta; b, primer set; c, gender, indicating no biases due to individual samples or PCR primer sets.

**Methods.** First trimester (8 - 9.5 weeks post-conception) and term placentas were collected at St. Mary's Hospital, Manchester under approval of the Central Manchester Local Ethics Committee. Tissue from first trimester and normal term placenta (n = 4, 2 male, 2 female) for each stage was homogenised in 1ml TRI Reagent (Sigma) and total RNA isolated. First strand cDNA was synthesised using 1μg total RNA in a 20 μl reaction using random hexamer priming and the High Capacity cDNA Reverse Transcription Kit (Applied Biosystems). PCR was performed using Pfu Ultra Hot Start DNA Polymerase (Stratagene, UK) in a 50 μl reaction and the resulting PCR products were analysed on a 1% agarose gel. Amplicons were gel extracted using a Qiagen Gel Extraction Kit (Qiagen, UK) and subcloned into the vector pSTBlue-1 and transformed into NovaBlue Singles competent cells (Novagen, UK). Colonies were picked and grown overnight in LB containing ampicillin at 50 μg/ml and plasmid DNA was extracted using a Qiagen spin mini-prep kit (Qiagen, UK). Plasmids were analysed for the presence of an insert and positive clones were sent for sequencing (GATC Biotech, Germany).

PCR primer sequences:

I1a F: 5’-AGAGACCATGGGAACCCTCT

I1a R: 5’-ATTCTGGATCAGCAGGGATG

I1b F: 5’-ACAGCGCATCAAATGGAAG

I1b R: 5’-AGCAGGGATGCATTGGAATA

Ex5 F: 5’-GACCTCCCCAGAATTTACCC

Ex5 R: 5’-TCATGGATTTGGAGCTTTCC

**Figure S2: PSG1-mediated inhibition of the platelet-fibrinogen interaction using platelets activated with 250 nM thromboxane mimetic U46619, 10 µM adenosine diphosphate (ADP) and 25 µM epinephrine. PSG1 significantly inhibited fibrinogen binding initiated by all three activators. Data are means of four independent experiments ± S.E.M. **, P <0.01; ***, P < 0.001, one-way ANOVA with Tukey’s multiple comparison post test of ‘Activated’ and ‘Activated & PSG1’ datasets.**


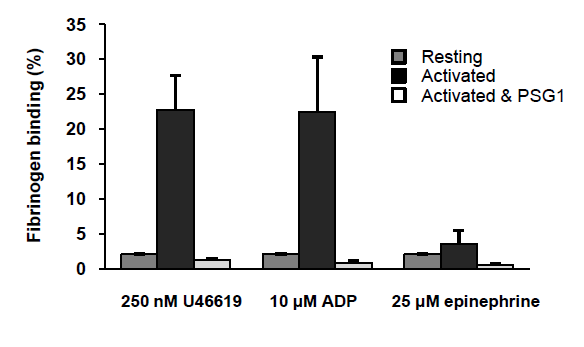


******

*******

******

**Figure S3: Amino acid sequences of all PSG protein variants used.**

V5-His (all human PSG variants) and MYC-His (mouse Psg23) carboxy-terminal affinity tags are underlined. Plasmids for each mutant were sequenced (GATC Biotech, Germany), and translated using the ExPASy protein translation tool. PSG domain structure as determined by the Simple Modular Architecture Research Tool (SMART) hosted by Uniprot at EMBL: [Schultz et al. (1998) Proc. Natl. Acad. Sci. USA 95,](http://www.ncbi.nlm.nih.gov/pubmed/18978020) 5857-5864; Letunic et al. (2009) Nucleic Acids Res., doi:10.1093/nar/gkn808.

= Signal Peptide (confirmed by N-terminal sequencing of PSG1 wildtype)

= N domains

= A1 domain

= A2 domain (A domain in PSG23)

= B2 domain

PSG1-KGD (wildtype)

MGTLSAPPCTQRIKWKGLLLTASLLNFWNLPTTAQVTIEAEPTKVSEGKDVLLLVHNLPQNLTGYIWYKGQMRDLYHYITSYVVDGEIIIYGPAYSGRETAYSNASLLIQNVTREDAGSYTLHIIKGDDGTRGVTGRFTFTLHLETPKPSISSSNLNPRETMEAVSLTCDPETPDASYLWWMNGQSLPMTHSLKLSETNRTLFLLGVTKYTAGPYECEIRNPVSASRSDPVTLNLLPKLPKPYITINNLNPRENKDVLNFTCEPKSENYTYIWWLNGQSLPVSPRVKRPIENRILILPSVTRNETGPYQCEIRDRYGGIRSDPVTLNVLYGPDLPRIYPSFTYYRSGEVLYLSCSADSNPPAQYSWTINEKFQLPGQKLFIRHITTKHSGLYVCSVRNSATGKESSKSMTVEVSDWTVPKLRPGKPIPNPLLGLDSTRTGHHHHHH

PSG1-RGE

MGTLSAPPCTQRIKWKGLLLTASLLNFWNLPTTAQVTIEAEPTKVSEGKDVLLLVHNLPQNLTGYIWYKGQMRDLYHYITSYVVDGEIIIYGPAYSGRETAYSNASLLIQNVTREDAGSYTLHIIKRGEGTRGVTGRFTFTLHLETPKPSISSSNLNPRETMEAVSLTCDPETPDASYLWWMNGQSLPMTHSLKLSETNRTLFLLGVTKYTAGPYECEIRNPVSASRSDPVTLNLLPKLPKPYITINNLNPRENKDVLNFTCEPKSENYTYIWWLNGQSLPVSPRVKRPIENRILILPSVTRNETGPYQCEIRDRYGGIRSDPVTLNVLYGPDLPRIYPSFTYYRSGEVLYLSCSADSNPPAQYSWTINEKFQLPGQKLFIRHITTKHSGLYVCSVRNSATGKESSKSMTVEVSDWTVPKLRPGKPIPNPLLGLDSTRTGHHHHHH

PSG1-AAA

MGTLSAPPCTQRIKWKGLLLTASLLNFWNLPTTAQVTIEAEPTKVSEGKDVLLLVHNLPQNLTGYIWYKGQMRDLYHYITSYVVDGEIIIYGPAYSGRETAYSNASLLIQNVTREDAGSYTLHIIAAADGTRGVTGRFTFTLHLETPKPSISSSNLNPRETMEAVSLTCDPETPDASYLWWMNGQSLPMTHSLKLSETNRTLFLLGVTKYTAGPYECEIRNPVSASRSDPVTLNLLPKLPKPYITINNLNPRENKDVLNFTCEPKSENYTYIWWLNGQSLPVSPRVKRPIENRILILPSVTRNETGPYQCEIRDRYGGIRSDPVTLNVLYGPDLPRIYPSFTYYRSGEVLYLSCSADSNPPAQYSWTINEKFQLPGQKLFIRHITTKHSGLYVCSVRNSATGKESSKSMTVEVSDWTVPKLRPGKPIPNPLLGLDSTRTGHHHHHH

PSG1ΔN

MGTLSAPPCTQRIKWKGLLLTASLLNFWNLPTTAETPKPSISSSNLNPRETMEAVSLTCDPETPDASYLWWMNGQSLPMTHSLKLSETNRTLFLLGVTKYTAGPYECEIRNPVSASRSDPVTLNLLPKLPKPYITINNLNPRENKDVLNFTCEPKSENYTYIWWLNGQSLPVSPRVKRPIENRILILPSVTRNETGPYQCEIRDRYGGIRSDPVTLNVLYGPDLPRIYPSFTYYRSGEVLYLSCSADSNPPAQYSWTINEKFQLPGQKLFIRHITTKHSGLYVCSVRNSATGKESSKSMTVEVSDWTVPKLRPGKPIPNPLLGLDSTRTGHHHHHH

PSG1ΔA1

MGTLSAPPCTQRIKWKGLLLTASLLNFWNLPTTAQVTIEAEPTKVSEGKDVLLLVHNLPQNLTGYIWYKGQMRDLYHYITSYVVDGEIIIYGPAYSGRETAYSNASLLIQNVTREDAGSYTLHIIKGDDGTRGVTGRFTFTLHPKLPKPYITINNLNPRENKDVLNFTCEPKSENYTYIWWLNGQSLPVSPRVKRPIENRILILPSVTRNETGPYQCEIRDRYGGIRSDPVTLNVLYGPDLPRIYPSFTYYRSGEVLYLSCSADSNPPAQYSWTINEKFQLPGQKLFIRHITTKHSGLYVCSVRNSATGKESSKSMTVEVSDWTVPKLRPGKPIPNPLLGLDSTRTGHHHHHH

PSG1ΔA2

MGTLSAPPCTQRIKWKGLLLTASLLNFWNLPTTAQVTIEAEPTKVSEGKDVLLLVHNLPQNLTGYIWYKGQMRDLYHYITSYVVDGEIIIYGPAYSGRETAYSNASLLIQNVTREDAGSYTLHIIKGDDGTRGVTGRFTFTLHLETPKPSISSSNLNPRETMEAVSLTCDPETPDASYLWWMNGQSLPMTHSLKLSETNRTLFLLGVTKYTAGPYECEIRNPVSASRSDPVTLNLLYGPDLPRIYPSFTYYRSGEVLYLSCSADSNPPAQYSWTINEKFQLPGQKLFIRHITTKHSGLYVCSVRNSATGKESSKSMTVEVSDWTVPKLRPGKPIPNPLLGLDSTRTGHHHHHH

PSGΔA1/A2

MGTLSAPPCTQRIKWKGLLLTASLLNFWNLPTTAQVTIEAEPTKVSEGKDVLLLVHNLPQNLTGYIWYKGQMRDLYHYITSYVVDGEIIIYGPAYSGRETAYSNASLLIQNVTREDAGSYTLHIIKGDDGTRGVTGRFTFTLHLYGPDLPRIYPSFTYYRSGEVLYLSCSADSNPPAQYSWTINEKFQLPGQKLFIRHITTKHSGLYVCSVRNSATGKESSKSMTVEVSDWTVPKLRPGKPIPNPLLGLDSTRTGHHHHHH

PSG1ΔB2

MGTLSAPPCTQRIKWKGLLLTASLLNFWNLPTTAQVTIEAEPTKVSEGKDVLLLVHNLPQNLTGYIWYKGQMRDLYHYITSYVVDGEIIIYGPAYSGRETAYSNASLLIQNVTREDAGSYTLHIIKGDDGTRGVTGRFTFTLHLETPKPSISSSNLNPRETMEAVSLTCDPETPDASYLWWMNGQSLPMTHSLKLSETNRTLFLLGVTKYTAGPYECEIRNPVSASRSDPVTLNLLPKLPKPYITINNLNPRENKDVLNFTCEPKSENYTYIWWLNGQSLPVSPRVKRPIENRILILPSVTRNETGPYQCEIRDRYGGIRSDPVTLNVLKLRPGKPIPNPLLGLDSTRTGHHHHHH

PSG1-N-KGD

MGTLSAPPCTQRIKWKGLLLTASLLNFWNLPTTAQVTIEAEPTKVSEGKDVLLLVHNLPQNLTGYIWYKGQMRDLYHYITSYVVDGEIIIYGPAYSGRETAYSNASLLIQNVTREDAGSYTLHIIKGDDGTRGVTGRFTFTLHLKLRPGKPIPNPLLGLDSTRTGHHHHHH

PSG1-N-AAA

MGTLSAPPCTQRIKWKGLLLTASLLNFWNLPTTAQVTIEAEPTKVSEGKDVLLLVHNLPQNLTGYIWYKGQMRDLYHYITSYVVDGEIIIYGPAYSGRETAYSNASLLIQNVTREDAGSYTLHIIAAADGTRGVTGRFTFTLHLKLRPGKPIPNPLLGLDSTRTGHHHHHH

PSG9

MGPLPAPSCTQRITWKGLLLTASLLNFWNPPTTAEVTIEAQPPKVSEGKDVLLLVHNLPQNLPGYFWYKGEMTDLYHYIISYIVDGKIIIYGPAYSGRETVYSNASLLIQNVTRKDAGTYTLHIIKRGDETREEIRHFTFTLYLETPKPYISSSNLNPREAMEAVRLICDPETLDASYLWWMNGQSLPVTHRLQLSKTNRTLYLFGVTKYIAGPYECEIRNPVSASRSDPVTLNLLPKLPIPYITINNLNPRENKDVLAFTCEPKSENYTYIWWLNGQSLPVSPGVKRPIENRILILPSVTRNETGPYQCEIRDRYGGLRSNPVILNVLYGPDLPRIYPSFTYYRSGENLDLSCFTESNPPAEYFWTINGKFQQSGQKLFIPQITRNHSGLYACSVHNSATGKEISKSMTVKVSGPCHGDLTESQSKLRPGKPIPNPLLGLDSTRTGHHHHHH

PSG23

MGVTSELFTNVLIPWQRVLFTASLLTCWLLSTTASVTIQSPQHVVEGENILLQVDNLPENLLAFAWYRGLTNWRLTIAVYLLDYSTSMTGPEHSDREILYSNGSLWIQNVTQEDTGYYTLQTISNHGELESNTSTFLQVYSSHFTCGRPSFPAKLTIESVPPSVAEGGSVLLRVHNLPEYLQLFFWYKGVIMIHKVEIVRYRTLKNLSDPGPAHSGREIVYSNGSLLLQNVTWKDTGFYTLQTVNRYWKMELAHIYLQVDTPCCDPLDSAQLRIDPVTPHAAEGESVLLQVHNLPEDLQTFSWYKGVDSTPSFQIVEYSKAMKSIISGSAYSRREIGYTNGSLLLQDVTEKDSGLYTLVTIDSNMRVETVHVQVNIYKLVTQPVMRVSETTVRVQSSVVFTCFSDNTGVSIRWLFNKQSLQLTERMSLSPSKCQLRIHTVRKEDGGEYQCEAFNPANSKTSLPVSLAVMNEEQKLISEEDLHHHHHH

**Figure S4. Negative controls for platelet - fibrinogen interaction assay (see Fig. 1 in main text and associated Methods)**

**Figure S4 Legend.** Eluates from Ni-NTA affinity purification column containing contaminating proteins but not PSG1, V5-His tag synthetic peptide, and rabbit IgG do not inhibit platelet - fibrinogen interactions. Ni-NTA elution fractions were pooled together, concentrated and dialysed as described for PSG1 in Methods. V5-His tag synthetic peptide (see Supplementary Fig. 3 for sequence) was used at similar protein concentrations to PSG1 dose inhibition experiments resulting in very high V5-His molar concentrations relative to PSG proteins. TRAP-activated platelets are taken as 100% and resting platelets as 0%. Values greater than 100% are due to variation between platelet preparations from individual donors. Data are represented as mean ± S.E.M for three independent experiments.

**Figure S5. Co-immunoprecipitation of full-length PSG1 and PSG1 N-domain proteins with the KGD→AAA mutation by platelet integrin *α*_IIb_*β*_3_.**

**Figure S5 Legend.** Platelet integrin α_IIb_β_3_ mediated pull-down of full-length recombinant PSG1 and PSG1 N-domain proteins containing the wildtype KGD or mutant AAA tri-peptide motifs in the protein N-domain F-G loop. Lane: 1, wildtype PSG1; lane 2, PSG1 with KGD→AAA; lane 3, PSG1 N domain; lane 4, PSG1 N domain with KGD → AAA; lane 5, no αIIbβ3 control. Yellow asterisk in lower panel marks position PSG1 N domain protein in lanes 4 & 5; these proteins appear to have variable glycosylation suggested by fuzzy banding on gel.
